# *N*-arachidonoyl glycine, an abundant endogenous lipid, potently drives directed cellular migration through GPR18, the putative abnormal cannabidiol receptor

**DOI:** 10.1186/1471-2202-11-44

**Published:** 2010-03-26

**Authors:** Douglas McHugh, Sherry SJ Hu, Neta Rimmerman, Ana Juknat, Zvi Vogel, J Michael Walker, Heather B Bradshaw

**Affiliations:** 1 The Department of Psychological and Brain Sciences, 1101 East 10th Street, Indiana University, Bloomington, IN 47405, USA; 2 The Gill Center for Biomolecular Science, 1101 East 10th Street, Indiana University, Bloomington, IN 47405, USA; 3 The Neurobiology Department, Weizmann Institute of Science, Rehovot, Israel; 4 The Adelson Center for the Biology of Addictive Diseases, Sackler Faculty of Medicine, Tel Aviv University, Israel

## Abstract

**Background:**

Microglia provide continuous immune surveillance of the CNS and upon activation rapidly change phenotype to express receptors that respond to chemoattractants during CNS damage or infection. These activated microglia undergo directed migration towards affected tissue. Importantly, the molecular species of chemoattractant encountered determines if microglia respond with pro- or anti-inflammatory behaviour, yet the signaling molecules that trigger migration remain poorly understood. The endogenous cannabinoid system regulates microglial migration via CB_2 _receptors and an as yet unidentified GPCR termed the 'abnormal cannabidiol' (Abn-CBD) receptor. Abn-CBD is a synthetic isomer of the phytocannabinoid cannabidiol (CBD) and is inactive at CB_1 _or CB_2 _receptors, but functions as a selective agonist at this G_i/o_-coupled GPCR. *N*-arachidonoyl glycine (NAGly) is an endogenous metabolite of the endocannabinoid anandamide and acts as an efficacious agonist at GPR18. Here, we investigate the relationship between NAGly, Abn-CBD, the unidentified 'Abn-CBD' receptor, GPR18, and BV-2 microglial migration.

**Results:**

Using Boyden chamber migration experiments, yellow tetrazolium (MTT) conversion, In-cell Western, qPCR and immunocytochemistry we show that NAGly, at sub-nanomolar concentrations, and Abn-CBD potently drive cellular migration in both BV-2 microglia and HEK293-GPR18 transfected cells, but neither induce migration in HEK-GPR55 or non-transfected HEK293 wildtype cells. Migration effects are blocked or attenuated in both systems by the 'Abn-CBD' receptor antagonist O-1918, and low efficacy agonists *N*-arachidonoyl-serine and cannabidiol. NAGly promotes proliferation and activation of MAP kinases in BV-2 microglia and HEK293-GPR18 cells at low nanomolar concentrations - cellular responses correlated with microglial migration. Additionally, BV-2 cells show GPR18 immunocytochemical staining and abundant GPR18 mRNA. qPCR demonstrates that primary microglia, likewise, express abundant amounts of GPR18 mRNA.

**Conclusions:**

NAGly is the most effective lipid recruiter of BV-2 microglia currently reported and its effects mimic those of Abn-CBD. The data generated from this study supports the hypothesis that GPR18 is the previously unidentified 'Abn-CBD' receptor. The marked potency of NAGly acting on GPR18 to elicit directed migration, proliferation and perhaps other MAPK-dependent phenomena advances our understanding of the lipid-based signaling mechanisms employed by the CNS to actively recruit microglia to sites of interest. It offers a novel research avenue for developing therapeutics to elicit a self-renewing population of neuroregenerative microglia, or alternatively, to prevent the accumulation of misdirected, pro-inflammatory microglia which contribute to and exacerbate neurodegenerative disease.

## Background

In normal brain, microglia possess a characteristic ramified morphology which facilitates continuous immune surveillance [1,2]. When the CNS is damaged or infected, microglia undergo a phenotypic shift, altering their shape and expressing receptors that recognize endogenous and exogenous chemoattractants [3]. Receptor-initiated signaling cascades enable microglia to execute rapid, directed migration towards affected tissue [4]. Depending on the molecular species encountered, altered gene expression further adjusts the microglial phenotype towards pro- or anti-inflammatory [5,6]. Directed microglial migration is a major CNS defense and provides for homeostatic maintenance and tissue repair. Dysregulation of migration and phenotype leads to excessive pro-inflammatory and cytotoxic responses implicated in several neurodegenerative diseases, including multiple sclerosis and Alzheimer's disease [7-11]. Despite their importance, the mechanisms controlling microglial migration and phenotype remain poorly understood.

Endogenous cannabinoid signaling regulates microglial migration via CB_2 _receptors and an unidentified GPCR, the 'abnormal cannabidiol' (Abn-CBD) receptor [12,13] (a.k.a. the 'endothelial anandamide' receptor or CB_x_). The pharmacology of endogenous and phytocannabinoids is complex; well documented pharmacological evidence supports multiple cannabinoid receptor subtypes. Two have been cloned, CB_1 _and CB_2_, whereas others discriminated using pharmacological and genetic tools remain to be identified at the molecular level [14-17]. The 'Abn-CBD' receptor is the most prominent of these receptors and has been implicated in endothelium-dependent vasodilation in isolated resistance vessels, haemodynamic responses and modulation of microglial, endothelial and glioma cell migration [12,13,15,16,18-21]. Its defining characteristics are: activation by two synthetic isomers of cannabidiol (CBD), Abn-CBD and O-1602, which are inactive at CB_1 _and CB_2 _[15,16,18]. Other agonists include anandamide (AEA) and 2-arachidonoyl glycerol (2-AG), but not palmitoyl ethanolamide (PEA) [13,18,22]. CBD and *N*-arachidonoyl serine (ARA-S) are very low efficacy agonists behaving as partial agonists/antagonists depending on receptor expression levels; whereas another CBD analogue, O-1918, and rimonabant act as antagonists, although rimonabant does so only moderately [13,17,19,23]. The receptor is G_i/o_-coupled and its activation stimulates p44/42 mitogen-activated protein kinase (MAPK) [13,19].

*N*-arachidonoyl glycine (NAGly) is an endogenous metabolite of AEA, differing by the oxidation state of the carbon β to the amido nitrogen - a modification that drastically reduces its activity at CB_1 _and CB_2 _[24]. A wealth of data demonstrates that NAGly triggers antinociceptive and anti-inflammatory activities [25]. Several parallel pathways have been described for its synthesis [25], it is hydrolyzed by fatty acid amide hydrolase (FAAH) [25], and is a high affinity ligand for G_i/o_-coupled GPR18 [26] and a partial agonist of G_q/11_-coupled GPR92 receptors [27].

Here, using the immortalized primary microglial cell line (BV-2) [28], which have been shown to retain most of the morphological, phenotypical and functional properties described for freshly isolated active microglial cells [13,28], we investigate the hypothesis that NAGly and Abn-CBD regulate microglial migration through GPR18; identifying GPR18 as the unknown 'Abn-CBD' receptor. We demonstrate that NAGly is the most potent pro-migratory lipid for BV-2 microglia described to date and its effects mimic those of Abn-CBD at the 'Abn-CBD' receptor. Our data support the hypothesis that GPR18 is the 'Abn-CBD' receptor and suggest that NAGly is a primary means for initiating directed microglial migration in the CNS.

## Results

### NAGly potently induces directed microglial migration

**Figure 1:**
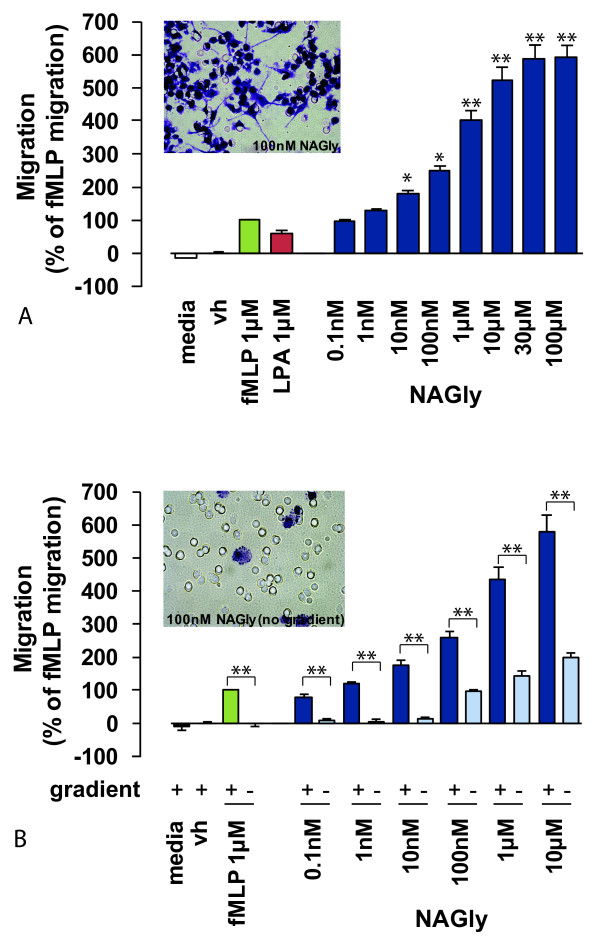
**NAGly-induced directed BV-2 microglial migration**. (A) BV-2 microglial migration in response to basal conditions; vh (0.1% DMSO); 1 μM fMLP; 1 μM LPA; 0.1 nM - 300 μM NAGly. * = P < 0.05, ** = P < 0.01 compared to 1 μM fMLP; one-way ANOVA; *n *= 8. Insert is a filter photograph of one random field of view at ×40 magnification indicating the migration produced by 100 nM NAGly. The 10 μm diameter pores can be discerned as the clear unstained circles. (B) BV-2 microglial migration in response to basal conditions; vh (0.1% DMSO); 1 μM fMLP ± concentration gradient; 0.1 nM - 10 μM NAGly ± concentration gradient. ** = P < 0.01 compared to the corresponding concentration gradient; Student's unpaired *t*-test; *n *= 3. Insert is a filter photograph of one random field of view at ×40 magnification indicating the migration produced by 100 nM NAGly in the absence of a concentration gradient.

Directed microglial migration and phenotypic modifications are known to be stimulated by factors including bacterial peptides, lysophospholipids and endocannabinoids [13,29,30]. Therefore, we compared NAGly-induced BV-2 microglial migration with *N*-formyl-methionine-leucine-phenylalanine (fMLP) and archidonoyl lysophophatidic acid (LPA), chemotactic ligands released under conditions of brain injury or infection [31,32]. NAGly potently induced concentration-dependent migration, and elicited a response twice that produced by 1 μM fMLP or LPA at NAGly concentrations of 0.17 nM and 0.08 nM, respectively (Figure 1A).

Chemotaxis (directed migration) is the process whereby cells sense soluble molecules and purposely advance along a concentration gradient to their source. This is in contrast to chemokinesis (stimulated random motion), where cells experience spontaneous cytoskeletal polymerization which prompts indiscriminate meandering. Checkerboard analysis offers a means to differentiate migratory behaviour between chemotaxis and chemokinesis, and is based on disrupting the concentration gradient of a pro-migratory ligand. Indiscriminate cell migration across a filter membrane will remain unaffected by the absence of a concentration gradient. Whereas directed cell migration is prevented by the absence of the guidance cue derived from the concentration gradient. Checkerboard analysis of NAGly revealed BV-2 microglia exhibit chemotaxis, and purposely advance towards the source of NAGly in a directed manner (Figures 1A &1B). A low basal level of chemokinesis was observed, which is the case with all established chemoattractants (Figure 1B).

As NAGly undergoes hydrolysis via FAAH to form AA and glycine [25], both of which are signaling molecules in their own right, we investigated whether the NAGly-induced response was due to its metabolism to either of these products. 1 μM NAGly produced a migratory response (% of fMLP migration) of 435.9% ± 36.9% compared to 22.4% ± 2.9% for AA, and -15.4% ± 5.4% for glycine; these values are significantly different (P < 0.001 compared to 1 μM NAGly; one-way ANOVA; *n *= 3). This indicates that neither AA nor glycine can account for the migratory response produced in BV-2 microglial cells by NAGly.

**Figure 2:**
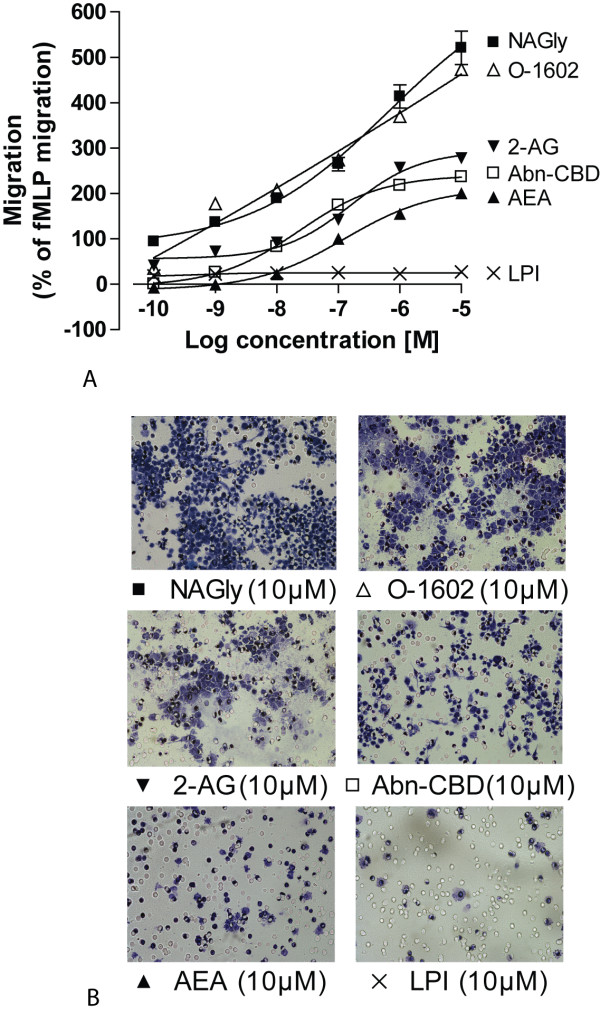
**NAGly-induced BV-2 microglial migration is concentration- and structure-dependent**. (A) BV-2 microglial migration in response to 0.1 nM - 10 μM concentrations of NAGly; AEA; 2-AG; PEA; PALGly; Abn-CBD; O-1602; LPI; *n *= 3. (B) Filter photographs of one random field of view at ×40 magnification indicating the migration produced by 10 μM concentrations of NAGly, O-1602, 2-AG, Abn-CBD, AEA and LPI.

In 2003, Walter *et al *described endocannabinoid system involvement in recruiting microglia toward dying neurons: pathological stimulation of neurons and microglia led to a dramatic and selective increase in 2-AG production which triggered microglial migration by engaging CB_2 _and 'Abn-CBD' receptors [13]. Therefore, we next compared NAGly-induced migration to various endocannabinoids, endogenous lipids and compounds relevant to 'Abn-CBD' receptor pharmacology to compare the potency of each of these compounds to induce migration (Figures 2A &2B). A response double that of 1 μM fMLP was elicited by the following concentrations of ligand: 0.17 nM NAGly, 0.27 nM O-1602, 5.2 nM 2-AG, 13.1 nM Abn-CBD and 123 nM AEA (Figure 2A). PEA (the endogenous AEA analogue), palmitoyl glycine (PALGly; the endogenous NAGly analogue), and *L*-α-lysophosphatidylinositol (LPI) all exerted a minimal, concentration-independent stimulation of migration. The mean migration achieved for these compounds across a concentration range of 0.1 nM - 10 μM being 12.5% ± 3.5% (PEA; *n *= 3), 16.9% ± 3.9% (PALGly; *n = *3), and 23.5% ± 4.7% (LPI; *n *= 3). Thus, NAGly potently induced concentration-dependent migration of BV 2 microglia, and was more efficacious than previously described cannabinoid ligands (Fig 2A). A ~50-fold greater concentration of 2-AG than NAGly was required to reach the half-maximal response of 2-AG; and in terms of AEA, a ~1000-fold greater concentration of AEA than NAGly was required to reach the half-maximal response of AEA.

**Figure 3:**
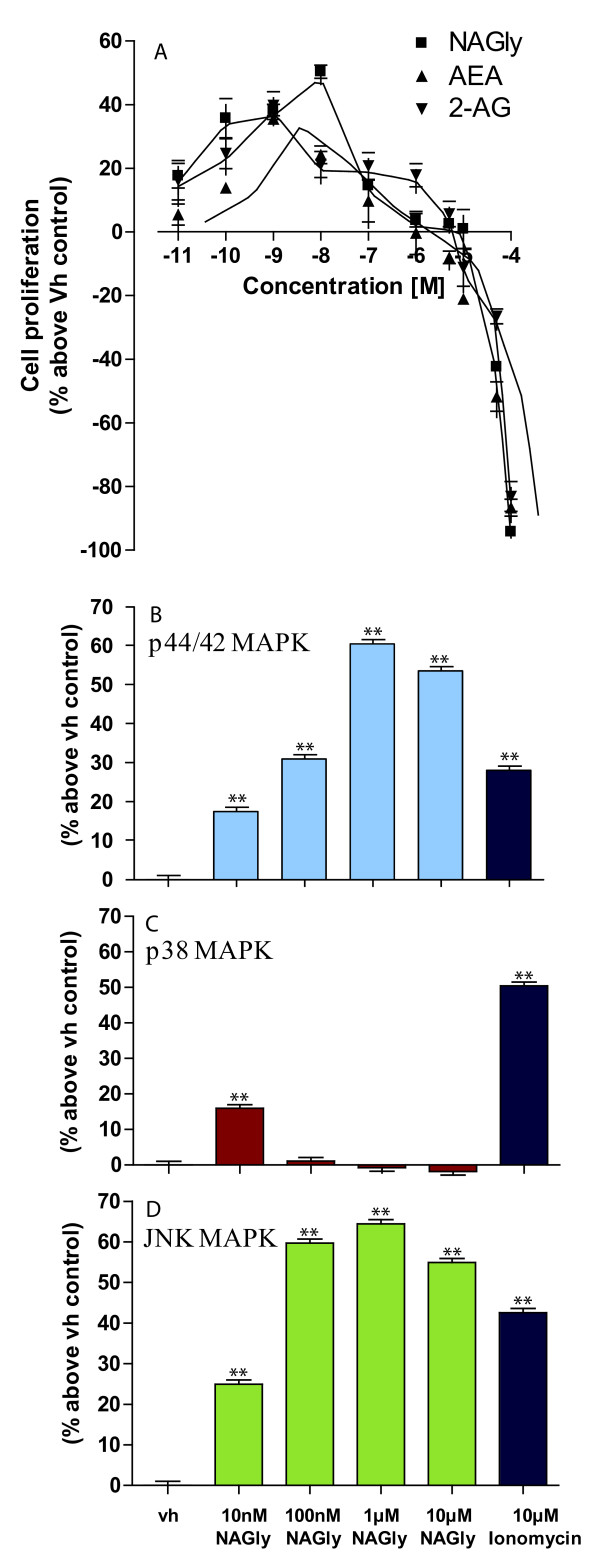
**NAGly-induced BV-2 cell proliferation and MAPK enzyme activation**. (A) BV-2 microglial proliferation in response to 0.01 nM - 100 μM concentrations of NAGly; AEA; 2-AG; *n *= 3. (B) p44/42 MAPK activation in BV-2 microglia in response to vh (0.1% DMSO) for 3 hours; 10 nM - 10 μM NAGly for 3 hours; 10 μM Ionomycin for 5 min. ** = P < 0.01 compared to vh; one-way ANOVA; *n *= 3. (C) p38 MAPK activation in BV-2 microglia in response to vh (0.1% DMSO) for 3 hours; 10 nM - 10 μM NAGly for 3 hours; 10 μM Ionomycin for 5 min. ** = P < 0.01 compared to vh; one-way ANOVA; *n *= 3. (D) JNK MAPK activation in BV-2 microglia in response to vh (0.1% DMSO) for 3 hours; 10 nM - 10 μM NAGly for 3 hours; 10 μM Ionomycin for 5 min. ** = P < 0.01 compared to vh; one-way ANOVA; *n *= 3.

Microglia in the adult CNS derive chiefly from a self-renewing population or rarely are replenished from adult bone marrow [31]. As they invade an injured region of the CNS, microglia can enter the cell cycle and proliferate via mitosis [5], e.g. elevated numbers of microglia are found in brains of patients with multiple sclerosis [33], Alzheimer's disease [34] and HIV [35]. The reduction of tetrazolium salts is widely accepted as a reliable way to examine cell proliferation. In the MTT reduction technique, the yellow tetrazolium 3-(4,5-dimethyl-thiazoyl-2-yl)-2,5-diphenyltetrazolium bromide (MTT) is reduced by metabolically active cells, in part by the action of dehydrogenase enzymes, to generate reducing equivalents such as NADH and NADPH. The resulting intracellular purple formazan dye can be solubilised and quantified by spectrophotometric means. Using this means to quantify cell proliferation in response to NAGly, AEA and 2-AG, we found that NAGly increased the population of BV-2 microglia at picomolar to low nanomolar concentrations after 24 hours (Figure 3A). The rank order of potency was NAGly > 2-AG > AEA at stimulating BV-2 cell proliferation: a ~50% increase was achieved by 10 nM NAGly, which was significantly greater than the ~24% and ~21% seen with 10 nM AEA and 2-AG, respectively (P < 0.01; one-way ANOVA; *n *= 3) (Figure 3A). Decreased cell viability was observed for all three compounds at concentrations greater than 1 μM. Carrier *et al *had previously shown that 2-AG, but not AEA, exerted a M-CSF (macrophage-colony stimulating factor) dependent proliferative effect on rat RTMGL1 microglia via CB_2 _receptors [36]. They observed ~30% increase with 300 nM 2-AG 24 hours after treatment, and this was accompanied by an increase in active p44/42 MAPK (a.k.a. ERK1/2). MAPKs respond to extracellular stimuli/mitogens and regulate activities such as cell proliferation, differentiation, motility, and death. As migration is an activated-MAPK-dependent phenomenon and 'Abn-CBD' receptors have been shown to induce p44/42 MAPK phosphorylation [13,19], we investigated the effect of NAGly on p44/42, p38 and JNK MAPK enzymes using In-Cell Western assays (Figures 3B, C &3D). NAGly induced a marked concentration-dependent phosphorylation of p44/42 and JNK MAPK (Figure 3B &3D), reflecting activation of these kinases, whereas, p38 MAPK was only significantly activated by 10 nM NAGly (Figure 3C). Our findings extend those of Carrier *et al*, showing that NAGly, 2-AG and AEA independently induce BV-2 microglial mitosis, with NAGly being the most potent of the three. Given the association between cell migration and proliferation, and that both are MAPK-dependent, 'Abn-CBD' receptor-activated phosphorylation of p44/42 and JNK MAPK in response to NAGly likely underlies the migratory and proliferative phenomena in BV-2 microglia.

In summary, the rank order of chemotactic potency published by Walter (2003) for BV-2 microglia was largely reproduced here. They found 2-AG > AEA > Abn-CBD at inducing migration, while PEA caused a weak concentration-independent response [13]. Here we report, NAGly > O-1602 > 2-AG > Abn-CBD > AEA, and the effects of PEA, LPI and PALGly were weak and concentration-independent (Figure 2A). NAGly is the most potent pro-migratory lipid for BV-2 microglia cells described to date, triggering directed migration and proliferation via MAPK activation (Figures 1, 2 &3).

### NAGly acts via a G_i/o_-coupled GPCR

**Figure 4:**
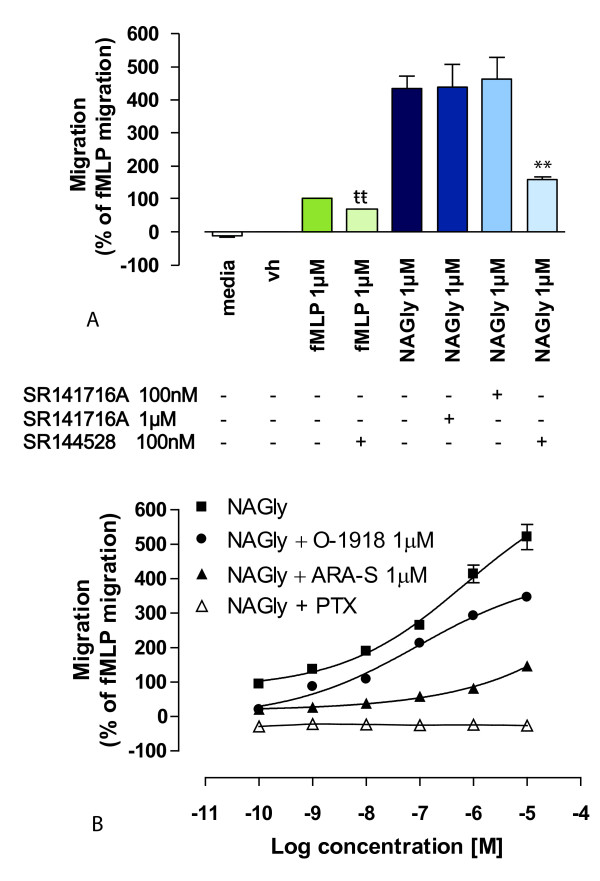
**NAGly-induced BV-2 microglial migration is G_i/o_-receptor mediated and can be antagonized**. (A) BV-2 microglial migration in response to basal conditions; vh (0.1% DMSO); 1 μM fMLP; 1 μM fMLP + 100 nM SR144528; 1 μM NAGly; 1 μM NAGly + 1 μM SR141716A; 1 μM NAGly + 100 nM SR141716A; 1 μM NAGly + 100 nM SR144528;. ** = P < 0.01 compared to 1 μM NAGly; tt = P < 0.01 compared to 1 μM fMLP; Student's unpaired *t*-test; *n *= 3. (B) BV-2 microglial migration in response to 0.1 nM - 10 μM NAGly ± 1 μM O-1918, ± 1 μM ARA-S, or ± 24 h pre-treatment with 1 μg/ml PTX; *n *= 3.

NAGly-stimulated BV-2 microglial migration was concentration- and structure-dependent (Figures 1A &2A), implying a receptor-mediated mechanism. In light of the known importance of engaging CB_2 _and 'Abn-CBD' receptors to trigger BV-2 migration [13], the relationship of these receptors to NAGly-induced BV-2 migration was investigated. The role of CB_1 _and CB_2 _receptors was examined using the antagonists/inverse agonists, rimonabant and SR144528. Rimonabant and SR144528 can block non-CB_1 _and non-CB_2 _targets when administered at concentrations greater than their K_d _values, i.e. in the micromolar range [13,36]. Therefore, 100 nM and 1 μM rimonabant was used with the aim of distinguishing between a CB_1 _or a non-CB_1 _rimonabant-sensitive eceptor; while 100 nM SR144528 was used to maintain CB_2 _selectivity. Neither concentration of rimonabant had an effect upon NAGly-induced migration, whereas SR144528 caused ~63.5% inhibition of the response to 1 μM NAGly (Figure 4A). However, NAGly does not demonstrate binding activity at either CB_1 _or CB_2 _receptors [25]. An alternative explanation is that SR144528 is exhibiting inverse agonism at constitutively active CB_2 _receptors [37] or blocking CB_2 _receptors involved in transactivation. This hypothesis is reasonable given that dual recruitment of CB_2 _and 'Abn-CBD' receptors is required for 2-AG-induced BV-2 migration [13], and SR144528 effects on constitutively active CB_2 _are observed in other migratory immune cells [17]. To test this, BV-2 microglia were pre-treated with 100 nM SR144528 before attempting to induce migration with 1 μM fMLP. fMLP is a tripeptide chemoattractant released from both bacteria and damaged mitochondria [38,39], and activates two formyl peptide receptors, designated FPR and FPRL-1 [40]. SR144528 caused ~32.2% inhibition of the response to 1 μM fMLP (Figure 4A). The estimated percentage viability ± 100 nM SR144528 was 97.3 ± 0.62 and 97.1 ± 0.67%, respectively; these values were not significantly different (P > 0.05; Student's unpaired *t-*test; *n *= 3), excluding cell death as a factor. Additionally, in subsequent experiments with HEK293 cells, which do not express CB_2 _receptors, we found that 100 nM SR144528 had no effect on HEK293 cells stably transfected with GPR18 induced in response to 1 μM NAGly; the migration being 497 ± 8 cells and 501 ± 11 cells in the presence and absence of SR144528 respectively (P > 0.05; Student's unpaired *t*-test; *n *= 3). These data instead infer a role for tonic CB_2 _signaling or transactivation in the migratory mechanism. Interactions among GPCRs are complex [41] and they have a propensity to experience cross-talk when co-expressed, e.g. receptor dimerization or heterologous desensitization. Thus, CB_2 _may cross-modulate with fMLP receptors and the G_i/o _receptor targeted by NAGly to regulate migration in BV-2 microglia. In summary, there is no evidence of a role for CB_1_, which is consistent with the low levels of CB1 gene product previously observed in BV-2 microglia [42]. While CB_2 _is demonstrably involved in BV-2 migration, it remains questionable that NAGly is signaling directly via CB_2 _receptors.

With regard to 'Abn-CBD' receptors, the agonist profile we observed with BV-2 migration was consistent with that known for this novel receptor, i.e. Abn-CBD, AEA, 2-AG and O-1602 stimulate migration (Figure 2A) [13,15,16,18,22,43]. However, 1 μM rimonabant failed to attenuated the NAGly response despite a reported IC_50 _value of 600 nM toward 'Abn-CBD' receptors (Figure 4A) [18]. Whether or not total block with 1 μM rimonabant should be expected in this circumstance would depend on the affinity of NAGly for the receptor, the concentration of NAGly employed and the number of 'Abn-CBD' receptors that need to be activated to see signaling; information that is not yet available. As a consequence, we further probed the role of 'Abn-CBD' receptors by investigating the antagonistic effects of ARA-S and O-1918 on NAGly- and fMLP-induced migration. In the presence of 1 μM ARA-S or 1 μM O-1918, the migration induced by NAGly was significantly attenuated (Figure 4B), whereas the migration in response to fMLP remained unaffected; 100.0% ± 3.5% (1 μM fMLP alone), 101.2% ± 2.9% (1 μM fMLP + 1 μM ARA-S), 100.8% ± 3.14% (1 μM fMLP + 1 μM O-1918). These values were not significantly different, P > 0.05; one-way ANOVA; *n *= 3. Likewise, neither ARA-S nor O-1918 had any effect on basal BV-2 cell migration; 0.0% ± 2.1% (Vh alone), 0.3% ± 1.9% (Vh + 1 μM ARA-S), 0.2% ± 2.3% (Vh + 1 μM O-1918). Similarly, these values were not significantly different, P > 0.05; one-way ANOVA; *n *= 3.

Since NAGly activates G_i/o_-coupled GPR18 and G_q/11_-coupled GPR92 [26,27], we investigated the effect of pertussis toxin (PTX) on the NAGly migratory response. PTX pre-treatment abolished the migration to NAGly (Figure 4B), without affecting cell viability. Using the trypan blue exclusion method, the estimated percentage viability of cells pre-treated for 24 hours was not different (± 1 μg/ml PTX was 97.8 ± 0.47 and 97.4 ± 0.51%, respectively; these values were not significantly different, P > 0.05; Student's unpaired *t-*test; *n *= 3). Taken together these data indicate G_i/o _GPCR involvement, and support the hypothesis that NAGly is acting via the 'Abn-CBD' GPCR to induce BV-2 microglial migration.

### BV-2 microglia express both GPR18 mRNA and GPR18 receptors

**Figure 5:**
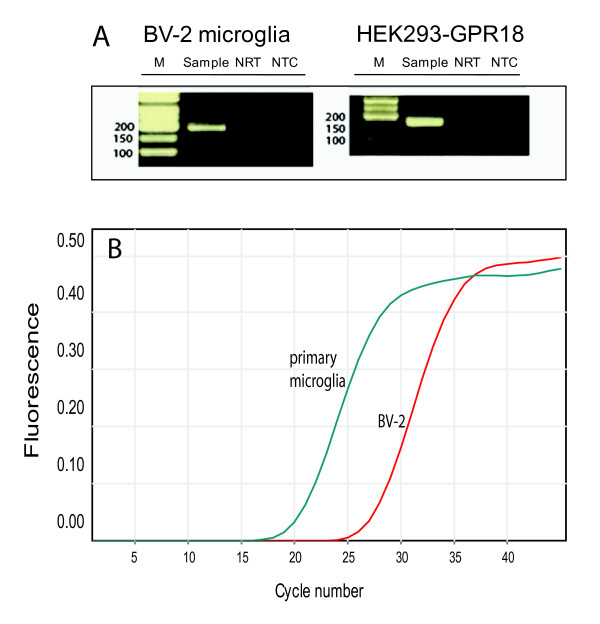
**BV-2 microglia express GPR18 mRNA and GPR18 receptors**. (A) Gel electrophoresis of BV-2 microglia and HEK293-GPR18 RT-qPCR products. RT-qPCR products were collected from the RT-qPCR run, loading buffer was added to the samples, and samples were run on a 2% agarose gel. No template control (NTC) and a control without reverse transcription (NRT) were used as controls. (B) Representative qPCR amplification curves showing the different amounts of mRNAs for GPR18 in primary microglia and BV-2 cells; *n *= 3.

**Figure 6:**
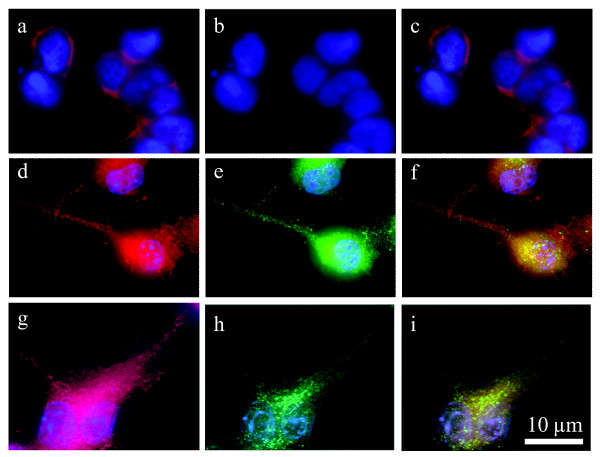
**BV-2 microglia and HEK293-GPR18 transfected, but not HEK293 wildtype, cells express GPR18**. Immunofluorescent confocal microscopy was conducted using an antibody against the GPR18 C-terminus (1:150; green), phalloidin to label actin (1:40; red), and DAPI (1.5 μg/ml) to label the nucleus (blue). *a*, HEK293 wildtype with DAPI and phalloidin. *b*, HEK293 wildtype with GPR18 antibody and phalloidin. *c*, HEK293 wildtype with GPR18 antibody, DAPI, and phalloidin. *d*, HEK293-GPR18 transfected with DAPI and phalloidin. *e*, HEK293-GPR18 transfected with GPR18 antibody and phalloidin. *f*, HEK293-GPR18 transfected with GPR18 antibody, DAPI, and phalloidin. *g*, BV-2 microglia with DAPI and phalloidin. *h*, BV-2 microglia with GPR18 antibody and phalloidin. *i*, BV-2 microglia with GPR18 antibody, DAPI, and phalloidin.

Our working hypothesis is that GPR18 is the 'Abn-CBD' receptor and that its activation by NAGly is a highly potent stimulation for microglial migration. For this to hold true, BV-2 microglia must express GPR18 receptors. Indeed, qPCR demonstrates that BV-2 and primary microglia express abundant amounts of GPR18 mRNA (Figures 5A &5B). In addition, immunocytochemical staining revealed GPR18 receptors are expressed in a heterogeneous punctuate pattern throughout BV-2 microglia and HEK293 cells stably transfected with GPR18, including their polymerized lamellipodia [44,45] (Figure 6). Lamellipodia are cytoskeletal actin protrusions on the mobile edge of a cell, believed to be both a steering device and the actual motor that pulls the cell forward during the process of chemotaxis [46-50]. Microglia adopt an amoeboid-like form and extend such motile lamellipodia, in order to achieve directed migration, enabling them to move toward relevant CNS locations and affect appropriate responses [51-54]. These data support our hypothesis that GPR18 mediates NAGly-induced directed migration of microglia.

### Overexpression of GPR18 affects directed migration induced by NAGly and Abn-CBD

**Figure 7:**
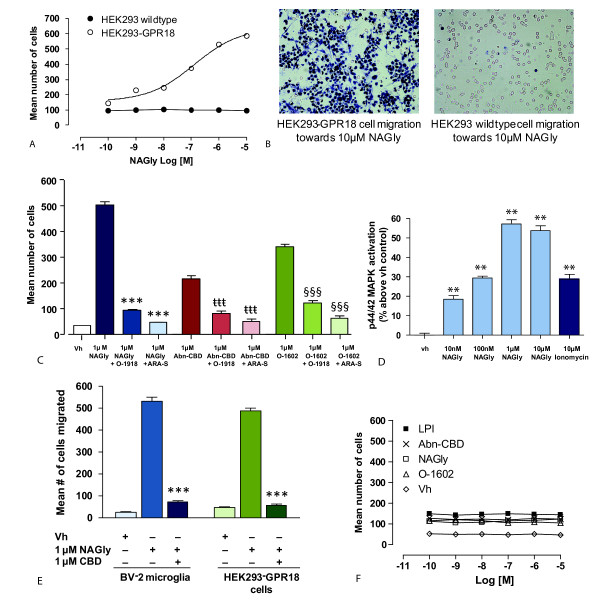
**NAGly-induced migration of HEK293-GPR18 and HEK293-GPR55 cells**. (A) HEK293 wildtype and HEK293-GPR18 transfected cell migration in response to 0.1 nM - 10 μM NAGly; *n *= 3. (B) Filter photographs of one random field of view at ×40 magnification indicating the migration produced by 10 μM concentrations of NAGly in HEK293-GPR18 and HEK293 wildtype cells. The 10 μm diameter pores can be discerned as the clear unstained circles. (C) HEK293-GPR18 cell migration in response to Vh (0.1% DMSO); 1 μM NAGly; 1 μM NAGly ± 1 μM O-1918; 1 μM NAGly ± 1 μM ARA-S. *** = P < 0.001 compared to 1 μM NAGly; one-way ANOVA; *n *= 3. HEK293-GPR18 cell migration in response to 1 μM Abn-CBD; 1 μM Abn-CBD ± 1 μM O-1918; 1 μM Abn-CBD ± 1 μM ARA-S; ttt = P < 0.001 compared to 1 μM Abn-CBD; *n *= 3. HEK293-GPR18 cell migration in response to 1 μM O-1602; 1 μM O-1602 ± 1 μM O-1918; 1 μM O-1602 ± 1 μM ARA-S. §§§ = P < 0.001 compared to 1 μM O-1602; one-way ANOVA; *n *= 3. (D) BV-2 microglia and HEK293-GPR18 cell migration in response to Vh (0.1% DMSO); 1 μM NAGly; 1 μM NAGly = 1 μM CBD; *** = P < 0.001 compared to 1 μM NAGly; one-way ANOVA; *n *= 3. (E) p44/42 MAPK activation in HEK293-GPR18 cells in response to vh (0.1% DMSO) for 3 hours; 10 nM - 10 μM NAGly for 3 hours; 10 μM Ionomycin for 5 min. ** = P < 0.01 compared to vh; one-way ANOVA; *n *= 3. (F) HEK293-GPR55 cell migration in response to 0.1 nM - 10 μM concentrations of LPI, Abn-CBD, NAGly, O-1602 and Vh (0.1% DMSO); *n *= 3.

To further examine the hypothesis that NAGly is acting through GPR18 to mediate its migratory effects in BV-2 microglia, and in light of there being no known GPR18 antagonists, we modelled BV-2 microglial migratory observations using wildtype or HEK293 cells stably transfected with HA11-tagged GPR18 (HEK293-GPR18). NAGly elicited a concentration-dependent migratory response in HEK293-GPR18 but not wildtype cells (Figures 7A &7B), with an E_max _similar to BV-2 microglia. 24 h pre-treatment with PTX abolished the response to 1 μM NAGly, the mean number of cells migrated with and without PTX pre-treatment was 505 ± 11 and 2 ± 1 respectively; these values were significantly different (P > 0.05; Student's unpaired *t*-test; *n *= 3). 1 μM NAGly-induced migration was also significantly attenuated in the presence of 1 μM O-1918 or 1 μM ARA-S (Figure 7C). Abn-CBD and O-1602 also induced migration in HEK293-GPR18 cells, with O-1602 being more potent than Abn-CBD (Figure 7C); both responses were significantly inhibited in the presence of 1 μM ARA-S or 1 μM O-1918 (Figure 7C), which again is in agreement with the BV-2 microglial data. CBD is known to behave as a partial agonist/antagonist of 'Abn-CBD' receptors depending on receptor expression levels [13,17]. 1 μM NAGly-induced migration of both BV-2 microglia and HEK293-GPR18 receptors was also significantly attenuated in the presence of 1 μM CBD (Figure 7D). The NAGly-induced p44/42 MAPK activation observed with BV-2 microglia too was reproduced in HEK293-GPR18 cells (Figure 7E).

Several publications have suggested the orphan receptor GPR55 interacts with certain cannabinoid ligands, including Abn-CBD and O-1602 [55]. While this proposition remains a contentious one, BV-2 microglia do express GPR55 mRNA [42]. Therefore we explored whether NAGly, Abn-CBD, O-1602 or LPI stimulate migration in HEK293 cells stably transfected with HA11-tagged GPR55 (HEK293-GPR55). All four of these compounds produced a weak, concentration-independent migratory response in HEK293-GPR55 cells (Figure 7F) that was irreconcilable with the NAGly, Abn-CBD and O-1602 effects on BV-2 migration.

## Discussion

'Abn-CBD' receptors have primarily been characterized in vascular tissue and microglia. Studies investigating the vasodilatory effects of AEA in CB_1_/CB_2 _knockout mice led to the postulation of the 'Abn-CBD' receptor as a novel endothelial cannabinoid target for which AEA, Abn-CBD and O-1602 were agonists that induced relaxation of the whole mesenteric arterial system [18]. Subsequent investigations have elaborated that multiple signaling pathways underlie the hemodynamic effects elicited by AEA, and involve CB_1_, TRPV1, 'Abn-CBD' receptors and perhaps another distinct endothelium-independent Abn-CBD/O-1602-sensitive target [22,56,57]. The specifics vary according to particular location in the vascular network and the preparation under scrutiny, e.g. aorta vs mesenteric artery segments, endothelium-intact vs endothelium-denuded vessels [18,19,22,58]. In 2003, well-executed studies with primary and BV-2 microglia reproduced the pharmacology of the endothelial 'Abn-CBD' receptor, revealing its expression and significant migratory role in microglia [13].

As a whole the cannabinoid field has eagerly awaited developments that will clarify the molecular identity of the 'Abn-CBD' receptor. Our analyses demonstrate that NAGly and Abn-CBD regulate cellular migration through GPR18, and we propose this GPCR is the unidentified 'Abn-CBD' receptor. Multiple lines of evidence substantiate this hypothesis: NAGly, at sub-nanomolar concentrations, together with the 'Abn-CBD' receptor agonists Abn-CBD and O-1602 [15,16,18], potently drives cellular migration in both BV-2 microglial and HEK293-GPR18 transfected cells, but not in HEK293-GPR55 or non-transfected HEK293 cells. O-1602 was ~50 times more effective than Abn-CBD at inducing migration in BV-2 microglia. This is in keeping with the work of Jarái *et al *which first characterized 'Abn-CBD' receptors in the rat mesenteric bed, where O-1602 was ~80 times more potent than Abn-CBD at causing vasodilation [18]. 'Abn-CBD' receptors couple via G_i/o _proteins [13]; here, PTX pre-treatment to uncouple such G_i/o _proteins prevented the migratory response to NAGly in BV-2 and HEK293-GPR18 cells. The NAGly-, Abn-CBD-, and O-1602-induced migration was blocked or attenuated in BV-2 or HEK293-GPR18 cells by the 'Abn-CBD' receptor antagonist O-1918, and low efficacy agonists ARA-S and CBD. NAGly promotes proliferation and activation of MAPK enzymes at low nanomolar concentrations in BV-2 cells and HEK293-GPR18 cells, demonstrating cellular responses correlated with microglial migration and previous 'Abn-CBD' receptor activity on p44/42 MAPK [13,19]. Finally, BV-2 microglia show heterogeneous GPR18 immunocytochemical staining, including the polymerized actin-containing lamellipodia that permit motile cells to achieve directed migration, and abundant GPR18 mRNA. qPCR demonstrates that primary microglia, likewise, express abundant amounts of GPR18 mRNA.

Both the academic community and pharmaceutical industry are engaged in intensive research of the endogenous cannabinoid signaling system, focussing on its potential therapeutic exploitation regarding mental illness, neuropathic and inflammatory pain, obesity, osteoporosis, nicotine addiction, cardiovascular disorders, and liver disease. Therefore, our recognition of GPR18 as the unidentified 'Abn-CBD' receptor has far-reaching implications. Firstly, hitherto unrecognized GPR18-mediated effects by cannabinoid ligands, particularly those that were previously classified as CB_1_- or CB_2_-receptor-selective, may have resulted in the misinterpretation of the role of those receptors in various systems. Secondly, our present definition and understanding of the endogenous cannabinoid signaling system will have to be expanded given the recognition of GPR18 as the 'Abn-CBD' receptor and that its endogenous ligand, NAGly, is a metabolic product of AEA [25]. Thirdly, elucidation of GPR18's other physiological roles will further reveal the molecular mechanisms responsible for the detrimental and medicinal effects of cannabis constituents. Lastly, GPR18-selective ligands will make available novel therapeutic routes targeting a broad spectrum of pathophysiologies.

With specific regard to the CNS, microglia represent a major cellular component of the brain, constituting a widely distributed network of immunoprotective cells [59,60]. During the last decades, it has become clear that the roles traditionally ascribed to microglia, i.e. to dispose of dead cells and debris and to mediate brain inflammatory states, are only a fraction of a much wider repertoire of functions spanning from brain development to aging and neuropathology [61,62]. Such functions are necessarily reliant upon the complex signaling systems subserving the reciprocal communication that occurs between neurons and microglia [63]. Indeed, the loss of specific communication between damaged neurons and microglia is viewed as responsible for the turning of microglia to a hyperactivated state, which allows them to escape neuronal control and to give rise to persistent inflammation, resulting in exacerbation of neuropathology [60].

## Conclusions

The marked potency of NAGly acting on GPR18 to elicit directed migration, proliferation and perhaps other MAPK-dependent phenomena advances our understanding of the lipid-based signaling mechanisms employed by the CNS to actively recruit microglia to sites of interest. It offers a novel research avenue for developing therapeutics to elicit a self-renewing population of neuroregenerative microglia, or alternatively, to prevent the accumulation of misdirected, pro-inflammatory microglia which contribute to and exacerbate neurodegenerative disease.

## Methods

### Cells in culture

The mouse microglial cell line BV-2 (a gift from Dr. N. Stella; University of Washington, Seattle), which was originally generated by immortalizing primary microglia (Blasi *et al*., 1990), were grown in high glucose DMEM (Gibco, USA) supplemented with FBS (10%), penicillin (100 units/ml), streptomycin (100 μg/ml), and passaged every 4-5 days for a maximum of 30 passages. HEK293 wildtype (ATCC, USA), HEK293 cells stably transfected with HA11-tagged GPR55 (HEK293-GPR55; previously generated [64]) and HEK293 cells stably transfected with HA11-tagged GPR18 (HEK293-GPR18; generated for this study), were grown in Eagle's MEM (Gibco, USA) supplemented with FBS (10%), penicillin (100 units/ml), streptomycin (100 μg/ml) and L-glutamine (0.292 mg/ml), and passaged every 4-5 days for a maximum of 30 passages. HEK293 cells were transfected with 2 μg of HA11-tagged hGPR18 plasmid using Lipofectamine and Plus reagents (Invitrogen, USA) in a 6-well plate using standard molecular biological techniques [65]. G418-resistant colonies were used as a positive control to validate the specificity of the hGPR18-CT purified antibody.

In order to obtain primary microglia for the RNA extraction studies, mixed glial cells were isolated from dissociated cerebral cortex of newborn (P0-P1) C57BL/6J mice as previously described [66]. The cell suspension was prepared in culture medium for glial cells [DMEM supplemented with 10% FCS, L-glutamine (1 mM), sodium pyruvate (1 mM), penicillin (100 U/ml), and streptomycin (100 mg/ml)] and cultured at 37°C/5% CO_2 _in 75-cm^2 ^Falcon tissue-culture flasks, coated with polyD-lysine (PDL). Half of the medium was changed after the first day and every second day thereafter, for a total culture time of 10-14 days. Microglia were shaken off the primary mixed brain glial cell cultures (150 rpm for 4-6 h at 37°C), with maximal yields between days 10 and 14. Cells were seeded onto PDL-pretreated 60 mm plates and grown in culture medium for microglia [RPMI medium supplemented with 10% FCS, L-glutamine (1 mM), sodium piruvate (1 mM), penicillin (100 U/ml) and streptomycin (100 mg/ml). The cells were allowed to adhere to the PDL-coated plate (30 min, 37°C/5% CO_2_) and the nonadherent cells were rinsed off. After 48 h microglial cells are ready to be used for experiments.

### Test compounds

Appropriate stock concentrations of the compounds tested in this study were prepared in 100% DMSO, before being serially diluted to achieve the desired final working concentrations, each containing 0.1% DMSO as vehicle. Lipid ligands were purchased from Enzo Life Sciences (Farmingdale, NY 11735) with the exception of O-1602, O-1918, and Abn-CBD, which were purchased from Cayman Chemicals (Ann Arbor, Michigan 48108).

### Migration assay

*In vitro *cell migration assays were performed using a modified 96-well Boyden Chamber and PVP-free polycarbonate filters with 10 μm diameter pores (Neuroprobe Inc., USA), which can be discerned as the clear unstained circles in the photographed filters of figures 1 &6. The upper wells of the Boyden chamber were filled with 50 μl of suspension of 1 × 10^6 ^cells ml^-1 ^in serum-free DMEM, before incubation with a 5% CO_2 _atmosphere at 37°C for 3 hours. 1 μM fMLP acted as positive control. Following incubation, non-migrated cells were then removed before fixation and staining with Diff-Quik^® ^stain set. Finally, the filter was sectioned and mounted onto microscope slides and the migrated cells counted in ten non-overlapping fields (×40 magnification) with a light microscope by multiple scorers blinded to experimental conditions. For inhibition of induced migration, cells were pre-incubated with antagonist for 30 min at 37°C in a water bath before loading into the upper wells, the lower wells contained the equivalent concentration of antagonist and test compound to ensure that the only concentration gradient present is that generated by the test compound as they diffuse through the pores in the filter.

### Cell proliferation assay

BV-2 cells were plated in 96-well plates at a seeding density of 1 × 10^4 ^cells per well overnight in media containing 1% FBS. The media was then changed to fresh media containing 1% FBS and the appropriate concentrations of test compound, then incubated for 24 hours. Cell density was assessed with the 3-(4,5-dimethyl-thiazoyl-2-yl)-2,5-diphenyltetrazolium bromide (MTT) formazan dye conversion assay (ATCC, USA) according to the manufacturer's instructions and measured at 570 nm with a SpectraMax M5 spectrophotometer (Molecular Devices, USA).

### In-Cell Western assay

An In-Cell Western assay was employed to simultaneously detect both the phosphorylated MAPK protein and normalize for total MAPK protein. The following primary antibodies were used to detect endogenous levels of the relevant total MAPK and phosphorylated MAPK: p44/42 MAPK rabbit pAb, and phospho-p44/42 MAPK mouse mAb (#4695 and #9106; Cell Signaling Technology, USA); p38 MAPK rabbit pAb, and phosphor-p38 MAPK mouse mAb (#9212 and #9216; Cell Signaling Technology, USA); and SAPK/JNK MAPK rabbit pAb, and phospho-SAPK/JNK MAPK mAb (#9252 and #9255; Cell Signaling Technology, USA).

BV-2 cells were plated into 96-well plates coated with 1 μg ml^-1 ^poly-L-lysine and treated with vehicle (Vh) (0.1% DMSO) or NAGly (10 nM - 10 μM) for 3 hours. Ionomycin (10 μM) treatment in the final 5 min was used as a positive control. Upon completion of the drug treatments, an In-cell Western assay was conducted: the 96-well plates were immediately placed on ice, the media removed and cells fixed with 100 μl/well of 3.7% formaldehyde in PBS for 15 min. The 96-well plates were then removed from the ice and allowed to warm up to room temperature over 30 min. The formaldehyde solution was replaced by 100 μl/well of ice-cold methanol and the plate kept at -20°C for 20 min. The cells were washed with 200 μl of 0.1% Triton X-100 in PBS with gentle shaking for 5 min at room temperature, the wash solution was removed before adding fresh 0.1% Triton X-100 and repeating for a total of 5 times. Following the final wash, cells were blocked with 150 μl of Odyssey blocking buffer (Li-Cor, USA) with moderate shaking for 90 min at 20°C. Primary antibody pairs (e.g. p44/42 and phospho-p44/42 MAPK) were diluted in Odyssey blocking buffer 1:200 and the plate was then incubated overnight with moderate shaking at 4°C. Primary antibody solution or Odyssey blocking buffer was then removed from all wells before they were washed with 0.1% Tween-20 in PBS with moderate shaking for 5 min at room temperature, this was repeated for a total of 5 times. Fluorescently labelled secondary antibodies (Odyssey 926-32211 goat anti-rabbit 800 nm antibody; 926-32220 goat anti-mouse 680 nm antibody; Li-Cor, USA) were diluted in Odyssey blocking buffer 1:800 containing 0.2% Tween-20. The secondary antibody solution was added to all wells and incubated in the dark for 90 min at room temperature. Secondary antibody solution was removed and the wells then washed with 0.1% Tween-20 in PBS with moderate shaking for 5 min at room temperature for a total of 5 times, while protecting from light. The final wash solution was removed and discarded. The plate was then scanned using the Li-Cor Odyssey Infrared Imaging System (Li-Cor, USA), using both 700 and 800 nm channels, a resolution of 42 μm, quality set to high, an intensity of 5, and focal offset of 4 mm.

Using the Odyssey application software, changes in MAPK activation were determined by calculating the mean background fluorescence from all non-primary antibody containing control wells, for both 700 and 800 nm channels. Background fluorescence was subtracted from the fluorescence measured in primary antibody containing wells, for both the 700 and 800 nm channels. The relative intensity of phospho-MAPK fluorescence was normalized against the relative intensity of fluorescence measured for total-MAPK. Finally the % response of all test compounds relative to vehicle was determined.

### Isolation of total RNA and real-time quantitative PCR (qPCR)

RNA was extracted from BV-2, HEK293-GPR18 transfected cells, and primary microglial cells using the RNAqueous^® ^small scale phenol-free total RNA isolation kit (Applied Biosystems, USA) and RNA samples (2 μg) were reverse transcribed using the SuperScript II™ Reverse Transcription Kit (Invitrogen, USA).

Expression of GPR18 mRNA in BV-2 and primary microglia was determined by RT-qPCR, using B2-MG as a normalizing gene, as previously described [67]. Normal, mock reversed transcribed samples (NRT), and no template controls (NTC; total mix without cDNA) were run for each of the examined mRNAs. RT-qPCR reactions were subjected to an initial HotStar Taq (Qiagen, USA) DNA polymerase activation step (15 min at 95°C), followed by 40 cycles each consisting of 15 s at 94°C, 30 s at 60°C and 30 s at 72°C. Fluorescence was measured at the end of each elongation step. Data were analyzed using the Rotor-gene software (Corbett Research, Australia) and a threshold cycle value Ct was calculated from the exponential phase of each RT-qPCR sample. Amounts of mRNA were calculated and expressed in relative units of SYBR Green fluorescence. PCR products were analyzed on a 2% agarose gel with ethidium bromide.

Expression of GPR18 in BV-2 microglia and HEK293-GPR18 cells was also determined by PCR using oligonucleotide primers based on the sequence of the *Mus musculus *G protein-coupled receptor 18 (GPR18) mRNA (GenBank Accession No. NM_182806.1) and B2-MG mRNA (GenBank Accession No. NM_009735). The primer sequences used were forward, TGAAGCCCAAGGTCAAGGAGAAGT and reverse, TTCATGAGGAA GGTGGTGAAGGCT (amplicon 163 bp) for the GPR18 and forward, ATGGGAAGCCGAACATACTG and reverse, CAGTCTCAGTGGGGGTGAAT (amplicon of 176 bp) for B2-MG. PCR reactions were subjected to an initial HotStart Taq DNA polymerase activation step of 95°C for 7 minutes, followed by 40 cycles of 94°C for 30 seconds, 55°C for 30 seconds, and 72°C for 30 seconds. PCR products were analyzed on a 2% agarose gel with ethidium bromide. Single bands corresponding to 163 bp for the GPR18 amplicon and 176 bp for the B2-MG amplicon were recorded.

### GPR18 antibody generation

A GST fusion protein expression construct was produced by inserting the DNA coding for a C-terminal 29-aa peptide (YRNYLRSMRRKSFRSGSLRSLSNINSEML) from human G-protein-coupled receptor (hGPR18) into a pGEX-3X vector at the BamH I and EcoR I restriction sites. The fusion protein was purified from BL21 *E. coli *lysates on a glutathione Sepharose column and was injected into two rabbits to generate antisera (Cocalico Biologicals, USA) using standard approaches [68]. The antiserum was purified in two steps, first by exclusion on a GST column and then by binding to and elution from an affinity column made with the injected GST fusion protein.

### Immunocytochemistry

**Figure 8:**
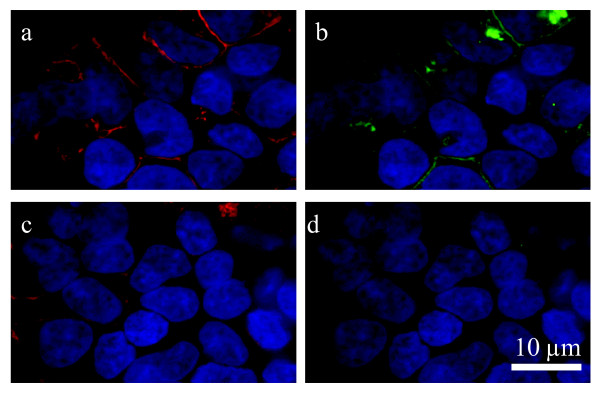
**GPR18 antibody recognizes hGPR18 receptors stably expressed in HEK293-GPR18 cells**. Immunofluorescent confocal microscopy was conducted using HEK293 cells stably transfected with HA11-tagged hGPR18 and HA11 (1:500) and GPR18 (1:500) antibodies. *a*, HA11 antibody (detected with Texas Red secondary; red) staining shows HA11-hGPR18 transfected cells. *b*, hGPR18 antibody (detected with FITC secondary; green) staining of the same cells identified with the HA11 antibody. *c*, HA11 antibody (detected with Texas Red secondary; red) staining shows HA11-hGPR18 transfected cells. *d*, hGPR18 antibody staining (detected with FITC secondary; green) was blocked when the GPR18 antibody was co-incubated with immunizing protein (30 μg/ml) for 1 hour before administration. DAPI (1.5 μg/ml; blue) was used to label the nucleus of all HEK293-GPR18 cells.

The GPR18 antibody generated for this study recognizes hGPR18 receptors stably expressed in HEK293-GPR18 cells (Figure 8) and endogenous GPR18 in BV-2 microglia (Figure 6). Cells were fixed with paraformaldehyde, blocked, and stained as follows: polyclonal rabbit anti-C-terminal GPR18 (1:150) (generated for this study) and Texas Red-conjugated phalloidin (1:40; Molecular Probes, Eugene, OR). Secondary IgG antibodies were FITC-conjugated donkey anti-rabbit (1:150; Jackson ImmunoResearch, USA). Images were acquired with a Nikon Eclipse TE2000-E confocal microscope (Nikon, USA).

### Analysis of data

For BV-2 microglia, the mean number of cells migrated in response to test compounds was normalized against the mean number of migrated cells elicited by 1 μM fMLP (0.1% DMSO). The number of migrated cells under vehicle only conditions (0.1% DMSO) was subtracted.

For HEK293 wildtype and HEK293-GPR18 transfected cells, simply the mean number of cells migrated above vehicle only conditions was used. All data are expressed as means ± s.e.mean and *n *= number of independent experiments. Statistical analyses were performed with GraphPad Prism 4. Concentration-response curves were generated using a sigmoidal dose-response (variable slope) curve-fitting process, except for that representing BV-2 cell proliferation where a simple point-to-point curve fit was employed instead.

## List of abbreviations

Abn-CBD: abnormal cannabidiol; ANOVA: analysis of variance; AA: arachidonic acid; AEA: *N*-arachidonoyl ethanolamine; 2-AG: 2-arachidonoyl glycerol; ARA-S: *N*-arachidonoyl-*L*-serine; B2-MG: beta-microglobulin; CBD: cannabidiol; CBN: cannabinol; CB_1_: cannabinoid receptor 1; CB_2_: cannabinoid receptor 2; CNS: central nervous system; DMEM: Dulbecco's Minimum Essential Medium; DMSO: dimethyl sulphoxide; ERK1/2: extracellular signal-regulated kinase 1/2; FAAH: fatty acid amide hydrolase; FBS: fetal bovine serum; fMLP: *N*-formyl-methionine-leucine-phenylalanine; GPCR: G protein-coupled receptor; LPA: arachidonoyl lysophosphatidic acid; LPI: *L*-α-lysophophatidylinositol; MAPK: mitogen-activated protein kinase; M-CSF: macrophage-colony stimulating factor; MTT 3-(4,5-dimethyl-thiazoyl-2-yl)-2,5-diphenyltetrazolium bromide; NAGly: *N*-arachidonoyl glycine; NRT: normal, mock reverse transcribed samples; NTC: total mix without cDNA; PTX: pertussis toxin; O-1602: *trans*-4- [3-methyl-6-(1-methylethenyl)-2-cyclohexen-1-yl]-5-methyl-1,3-benzenediol; O-1918: 1,3-dimethoxy-5-methyl-2- [(1R,6R)-3-methyl-6-(1-methylethenyl)-2-cyclohexen-1-yl)-benzene; PALGly: palmitoylglycine; PEA: palmitoyl ethanolamine; Rimonabant (a.k.a. SR141716A), *N*-(piperidin-1-yl)-5-(4-chlorophenyl)-1-(2,4-dichlorophenyl)-4-methyl-1*H*-pyrazole-3-carboximide hydrochloride; RT-PCR: reverse transcriptase polymerase chain reaction; SR144528: 5-(4-chloro-3-methylphenyl)-1-[(4-methylphenyl)methyl]-*N*--[(1*S*,4*R*,6*S*)-1,5,5-trimethyl-6bicyclo[2.2.1]heptanyl]pyrazole-3-carboxamide.

## Competing interests

The authors declare that they have no competing interests.

## Authors' contributions

DM performed the cell culture procedures, cell migration studies, cell proliferation experiments, In-Cell Western assays and immunocytochemistry imaging; design and coordination of the studies; data interpretation; statistical analyses; and manuscript preparation. NR PCR studies. AJ PCR studies. SSH generated the hGPR18 antibody; immunocytochemistry imaging. ZV PCR studies. JWM initial study design. HBB design and coordination of the studies, and manuscript preparation. All authors read and approved the final manuscript.
